# Distilling knowledge from multiple foundation models for zero-shot image classification

**DOI:** 10.1371/journal.pone.0310730

**Published:** 2024-09-20

**Authors:** Siqi Yin, Lifan Jiang

**Affiliations:** 1 School of Computer Science and Technology, Shandong University of Science and Technology, Qingdao, Shandong, China; 2 School of Computer Science and Technology, Shandong University of Science and Technology, Qingdao, Shandong, China; Guangdong University of Petrochemical Technology, CHINA

## Abstract

Zero-shot image classification enables the recognition of new categories without requiring additional training data, thereby enhancing the model’s generalization capability when specific training are unavailable. This paper introduces a zero-shot image classification framework to recognize new categories that are unseen during training by distilling knowledge from foundation models. Specifically, we first employ ChatGPT and DALL-E to synthesize reference images of unseen categories from text prompts. Then, the test image is aligned with text and reference images using CLIP and DINO to calculate the logits. Finally, the predicted logits are aggregated according to their confidence to produce the final prediction. Experiments are conducted on multiple datasets, including MNIST, SVHN, CIFAR-10, CIFAR-100, and TinyImageNet. The results demonstrate that our method can significantly improve classification accuracy compared to previous approaches, achieving AUROC scores of over 96% across all test datasets. Our code is available at https://github.com/1134112149/MICW-ZIC.

## 1 Introduction

In recent years, deep learning technologies have made significant progresses, particularly in the field of image classification, with numerous successful models being developed. For instance, ResNet [1] introduced a residual learning mechanism to address the difficulties in training deep networks, substantially increasing classification accuracy. Meanwhile, MobileNet [2] achieved efficient computation on mobile and embedded devices with its lightweight deep separable convolution design. Despite the significant success, these methods are built upon an assumption that the categories of the test images have been seen in the training set, which imposes restrictions in their practical applications. In real-world scenarios, we often encounter a large number of new categories that may not be included in the original training set.

To overcome the limitations of the aforementioned methods when dealing with unseen categories, zero-shot learning has garnered increasing attention in recent years, which aims to enable models to recognize categories that they have never seen during the training phase. Several prominent zero-shot models have been developed from various aspects. For example, an early zero-shot method [3] improved the classification accuracy of unseen bird species by learning the mapping between visual features and textual descriptions. Due to the relatively small number of images for training models, these methods still face challenges in achieving satisfactory zero-shot generalization capabilities.

In this paper, we propose a novel framework to leverage the alignment of images with text as well as the alignment among images themselves to further improve the zero-shot accuracy using multiple foundation models like ChatGPT, CLIP and DINO. Specifically, we first employ ChatGPT and DALL-E to generate images of unseen categories from text prompts. Then, the test image is aligned with text and reference images using CLIP and DINO to calculate the logits. Finally, the predicted logits are aggregated according to their confidence to produce the final prediction. To evaluate the effectiveness of our method, we conduct experiments on multiple datasets including CIFAR-10, CIFAR-100, and TinyImageNet. In comparison to previous zero-shot methods, our model produces significant gains in classification accuracy, with improvements of 11.97%, 26.06%, and 12.6% on CIFAR-10; 24.48%, 42.48%, and 5.42% on CIFAR-100; and 32.32%, 41.96%, and 1.2% on TinyImageNet, respectively. Furthermore, our model produces over 96% in terms of AUROC across all test datasets, particularly exceeding 99% on the CIFAR-10 dataset. These results clearly demonstrate the superiority of our method in zero-shot image classification.

## 2 Related work

### 2.1 Zero-shot classification

Zero-shot classification (ZSC) is a machine learning paradigm aimed at enabling models to recognize categories they have not seen during the training phase. Unlike traditional supervised learning methods, ZSC does not rely on direct experience with every class but rather achieves classification through understanding shared knowledge or attributes among categories [4–8]. This capability is crucial for dealing with data scarcity or category diversity issues, especially in fields such as Natural Language Processing (NLP) and Computer Vision (CV). In recent years, significant progress has been made in the field of ZSC. In particular, pre-trained language models such as GPT-3 [9] and BERT [10] show great potential in ZSC tasks. These models are trained on massive text datasets to learn rich linguistic features and world knowledge, which enables them to infer unknown categories without explicit examples. Additionally, by operating on graph-structured data, GNNs is able to capture complex relationships and attributes between categories, offering new avenues for accurate classification of unseen classes [11, 12]. To further improve zero-shot performance, Deep Calibration Network (DCN) [13] is developed to reduce the uncertainty of models when dealing with unseen categories. In addition, meta-learning has been studied to facilitate models to achieve higher accuracy on unseen images [14]. Moreover, a framework based on Generative Adversarial Networks (GANs) is proposed in [15], which can generate features representative of unseen categories to achieve further gains. To address the data imbalance issue in zero-shot classification, Balanced Meta-Softmax [16] is developed to achieve a better balance between different categories. Recent advancements such as [17], which refines the alignment between semantic and visual spaces, and [18], which improves disentangling cluster-based semantic representations, have further enhanced ZSC performance.

With the rapid advancements of recent foundation models, numerous attempts have been made to study these models from the perspective of zero-shot classification. CLIP [19] has demonstrated its superior performance across multiple computer vision benchmarks, particularly showing promising accuracy in zero-shot classification tasks. The success of CLIP highlights the potential of learning visual tasks with language supervision. Motivated by this, several attempts [20–24] have been made to adopt CLIP to classify images of unseen categories and produce promising results. For example, models like Calip [24] and ZegCLIP [23] leverage cross-modal joint learning to achieve more accurate classification of unseen categories by better aligning visual and textual representations. However, while these methods improve classification accuracy, they still face challenges in fully capturing the nuances of diverse and complex unseen categories, particularly in scenarios with limited or noisy data. Other methods [25–27] employ DINO to leverage the superior generalization capability of its features to boost downstream tasks like image classification. Recently, generative foundation models like DALL-E are also investigated for the zero-shot classification task. Specifically, Zhang *et al*. [28] employ generative models to produce additional images to augment the training dataset to achieve accuracy gains in zero-shot classification.

Despite superior performance of the aforementioned methods, the rich knowledge in foundation models are not fully exploited. To remedy this, in this paper, we propose to leverage foundation models from two perspectives to make full use of their knowledge. First, we employ ChatGPT and DALL-E to synthesize reference images that precisely describe unseen categories and classification boundaries, obtaining reference samples for new categories. Then, CLIP and DINO are employed to align the test image with reference images in the representation space to conduct image-image alignment. Meanwhile, CLIP is also adopted to associate the test image with text prompts for text-image alignment. Through these two kinds of alignment, the knowledge of foundation models can be better exploited to produce higher accuracy. Note that, different from [28] that uses generative models to produces additional samples for data augmentation, we employ generative models to synthesize reference images that are usually confused in appearance. As a result, our method achieves significant performance gains as compared to previous approaches, producing an increase of 0.45%, 0.24%, and 0.23% in classification accuracy on the CIFAR10, CIFAR100, and TinyImageNet datasets, respectively.

## 3 Method

**Fig 1:**
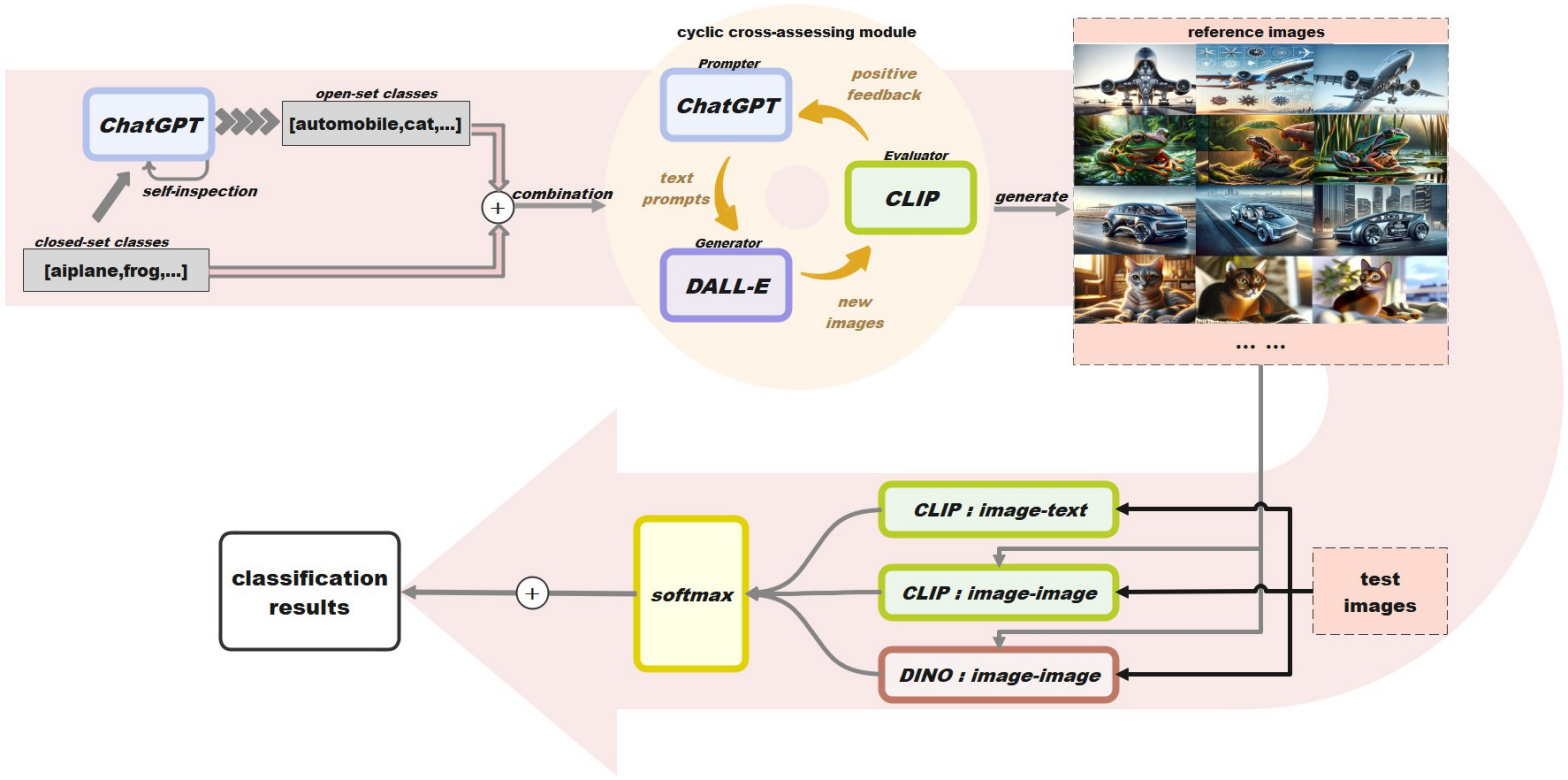
Overview of our method.

The overview of our method is summarized in Fig 1. Since GPT [29, 30] demonstrates its excellent capabilities in text generation and question answering, it is employed for text understanding. Inspired by the remarkable performance of DALL-E [31], it is adopted for image generation in our framework. To achieve text-image alignment, CLIP [19] is used due to its powerful capability. To extract distinct representation from the image, DINO [25] is selected due to its superior performance. Assume we have a dataset (*D*) containing *m* seen classes and *n* unseen classes. When conducting an image classification task on the dataset *D*, we first generate reference images for each category. Then, the test image *x* is aligned with class-definition text using CLIP to calculate the logits. Meanwhile, the test image is also aligned with reference images using CLIP and DINO to obtain the logits. Finally, the resultant logits are aggregated according to their confidence to obtain the final prediction.

### 3.1 Reference images generation

To synthesize images of unseen categories, ChatGPT and DALL-E are employed to leverage their rich knowledge.

Note that, several categories are easily confused in appearance since they may be similar in shape or features. Thus, we propose to generate reference images that are located near the boundaries of ambiguous categories. Specifically, we first conduct feature analysis to obtain common features of similar categories. Then, we synthesize images based on the resultant common features.

#### (1) Feature analysis

**Fig 2:**
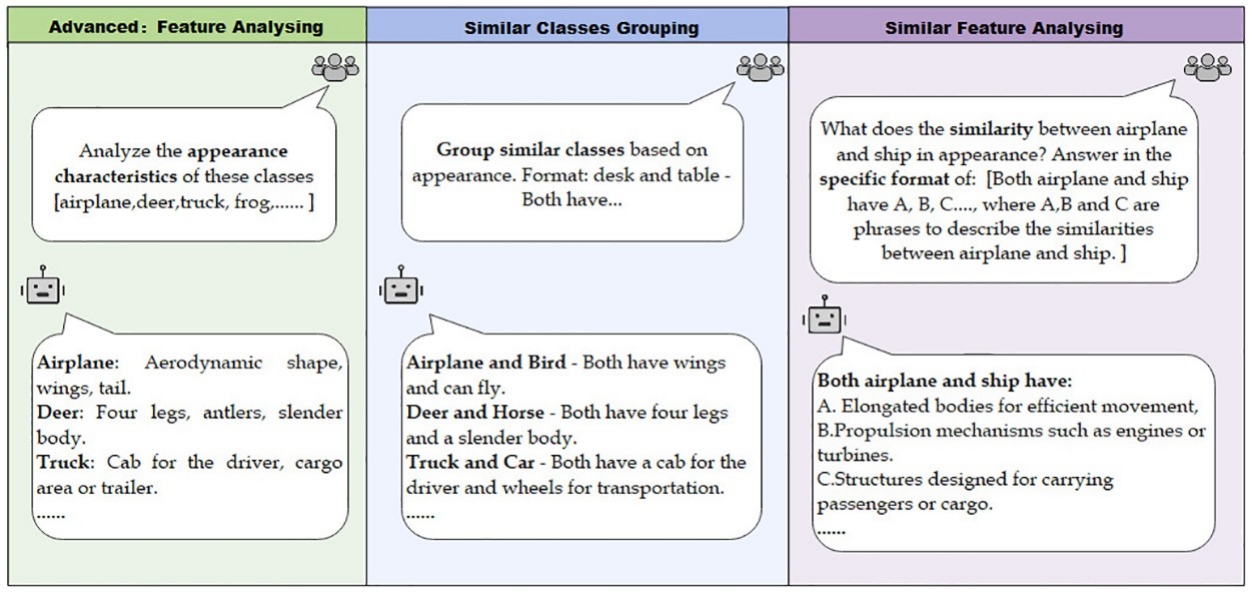
Examples of utilizing ChatGPT to generate common features of similar categories.

First, ChatGPT is adopted to analyze the appearance of *m* + *n* categories in the dataset, as shown in the left column of Fig 2. Then, ChatGPT is used to group categories that are similar in appearance. Specifically, we ask ChatGPT to identify those similar categories that are easily confused. Next, for each pair of resultant groups, ChatGPT is further adopted to analyze their common appearance features. Finally, the common features are obtained and denoted as *F*.

#### (2) Image synthesis

After feature analysis, DALL-E is employed to generate images of each class that possess the common appearance features *F*. For categories that do not resemble other categories, we directly utilize DALL-E to generate images belonging to it without using detailed appearance descriptions. To acquire more comprehensive reference information, we generate multiple images for each category. All these images will be employed in the subsequent steps for image-image alignment.

### 3.2 Alignment-driven image classification

To achieve accurate classification of an unseen test image, we align the image with text prompt and reference images using foundation models to distill their knowledge.

#### 3.2.1 Text-image alignment

CLIP is capable of encoding image and text into a shared representational space. With this property, the logits of the test image *x* and the unseen category *t*_*n*_ can be obtained by encoding *x* and *t*_*n*_ using CLIP and calculating the cosine similarity *S* of the resultant representations:

$$\begin{array}{c} S_{i-t,clip}^{n}=\text{cos}(F_{i}\left(x\right),F_{t}\left(t_{n}\right)), \end{array}$$ (1)

where *F*_*i*_(⋅) and *F*_*t*_(⋅) represent the image encoder and text encoder of CLIP.

#### 3.2.2 Image-image alignment

In addition to text-image alignment, we also conduct image-image alignment to leverage powerful vision foundation models. Specifically, the test image and the reference images are first encoded using CLIP and DINO to obtain their representations. Then, the cosine similarities between the resultant representations are calculated:

$$\begin{array}{c} S_{i-i,clip}^{n}=\frac{1}{M}\sum_{i=1}^{M}\;\text{cos}(F_{i}\left(x\right),F_{i}\left(r_{n}\right)), \end{array}$$ (2)

$$\begin{array}{c} S_{i-i,dino}^{n}=\frac{1}{M}\sum_{i=1}^{M}\;\text{cos}(D\left(x\right),D\left(r_{n}\right)), \end{array}$$ (3)

where *D*(⋅) refers to the image encoder of DINO, *r*_*n*_ denotes the *n*^*th*^ reference image.

### 3.3 Logits aggregation

After text-image and image-image alignment, a confidence-based strategy is employed to aggregate the resultant logits. That is, the logits with higher confidence are assigned with larger weights to produce the final results. Next, the final output is obtained as:

$$\begin{array}{c} S_{fuse}=W_{i-t,clip}\cdot S_{i-t,clip}+W_{i-i,clip}\cdot S_{i-i,clip}+W_{i-i,dino}\cdot S_{i-i,dino}. \end{array}$$ (4)

The confidence coefficient *W* is calculated using the following three options, which will be studied through ablation experiments in Sec. 4.3.

(1) Using the maximum value of each *S* as the confidence level:

$$\begin{array}{c} W=\text{max}{\left\{S^{n}\right\}}_{n=1}^{N}. \end{array}$$ (5)

(2) Using the inverse of the entropy as the confidence value:

$$\begin{array}{c} W=\frac{1}{H+10^{-6}},\;\;\;\;\;H=-\sum_{n}^{N}\;S^{n}\;\text{log}\left(S^{n}\right). \end{array}$$ (6)

where 10^−6^ is employed to avoid the denominator to be 0.

(3) Using the negative exponential of the entropy as the confidence value:

$$\begin{array}{c} W=\text{exp}(-H),\;\;\;\;\;H=-\sum_{n}^{N}\;S^{n}\;\text{log}\left(S^{n}\right). \end{array}$$ (7)

## 4 Experiments

### 4.1 Datasets and evaluation metrics

To evaluate the effectiveness of our proposed method, we conduct experiments on five widely-used datasets following previous protocol [32–34], including MNIST, SVHN, CIFAR10, CIFAR100, and TinyImageNet. For MNIST and SVHN and CIFAR10, we all select 6 categories as the closed set and the remaining 4 categories as the open set. For the CIFAR+N experiments, 4 classes are randomly sampled as the closed-set classes and N non-overlapping classes are randomly sampled from CIFAR100 as the open-set classes for evaluation (N = 10 for CIFAR-10 and N = 50 for CIFAR+50). For TinyImageNet, we select 20 categories as the closed set, with the other 180 categories as the open set. We employ Accuracy (AC) and Area Under the Receiver Operating Characteristic curve (AUROC). The AUROC metric is used to evaluate the model’s ability to differentiate between closed-set and open-set samples, that is, the accuracy of the model in identifying novel category samples.

### 4.2 Main results

We compare our method with nine state-of-the-art methods, including RPL [35], OpenHybrid [34], PMAL [36], ZOC [37], MLS [38], DIAS [39], Class-iinclusion [40], ODL [41], and CSSR [42]. Quantitative results are presented in Table 1. Compared with previous open-set classification methods, our method produces significant accuracy gains, especially on CIFAR-10 and TinyImageNet. For example, our method outperforms Class-inclusion by over 5% and 3% on CIFAR-10 and TinyImageNet, respectively. The higher accuracy of our method clearly demonstrate its effectiveness and superiority.

**Table 1:** AUROC results achieved by different methods.

Method	MNIST	SVHN	CIFAR10	CIFAR+10	CIFAR+50	TinyImageNet
**Methods that involve a training process**						
RPL [35]	91.7	93.1	90.1	97.6	96.8	80.9
OpenHybrid [34]	99.5	94.7	95.0	96.2	95.5	79.3
PMAL [36]	**99.7**	97.0	95.1	97.8	96.9	83.1
ZOC [37]	/	/	93.0	97.8	97.6	84.6
MLS [38]	99.3	97.1	93.6	97.9	96.5	83.0
DIAS [39]	99.2	94.3	85.0	92.0	91.6	73.1
Class-inclusion [40]	/	95.6	94.8	95.7	95.7	78.5
ODL [41]	99.5	94.3	85.7	89.1	88.3	76.4
ODL+ [41]	99.6	95.4	88.5	91.1	90.6	74.6
CSSR [42]	/	**97.9**	91.3	96.3	96.2	82.3
RCSSR [42]	/	97.8	91.5	96.0	96.3	81.9
**Methods that involve no extra training process**						
Ours	**99.7**	97.8	**99.8**	**98.4**	**97.8**	**96.4**

Table 2 shows the performance of our method compared to other methods across CIFAR10, CIFAR100, and TinyImageNet datasets. Our method achieves the best results in all metrics, including Top1, Top3, Top5 accuracy, and AUROC. Specifically, on CIFAR10, our method reaches **91.96%** in terms of Top1 accuracy and **99.78%** on AUROC. Similarly, it delivers the highest performance on CIFAR100 and TinyImageNet datasets.

**Table 2:** Performance comparison of single methods and our three-method fusion across CIFAR10, CIFAR100, and TinyImageNet datasets.

Method	CIFAR10				CIFAR100				TinyImageNet			
	Top1	Top3	Top5	AUROC	Top1	Top3	Top5	AUROC	Top1	Top3	Top5	AUROC
CLIP_text	79.99%	93.22%	97.41%	97.26%	47.69%	66.14%	72.35%	91.41%	41.20%	61.16%	69.24%	85.95%
CLIP_image	65.90%	86.20%	86.20%	98.12%	30.43%	48.55%	56.67%	85.73%	31.56%	48.17%	55.57%	82.68%
DINO	79.36%	90.48%	94.51%	97.97%	66.75%	81.77%	86.48%	94.28%	72.32%	72.32%	**86.79%**	96.07%
**Ours**	**91.96%**	**98.14%**	**99.30%**	**99.78%**	**72.17%**	**86.55%**	**90.36%**	**96.03%**	**73.52%**	**84.95%**	88.55%	**96.48%**

### 4.3 Ablation study

#### 4.3.1 Effectiveness of different components

We first conduct experiments to study the effectiveness for different components of our method, including image-text alignment using CLIP (M1), image-image alignment using CLIP (M2)m and image-image alignment using DINO (M3). Specifically, we compare the performance of our method with different combinations of components. As shown in Tables 3 and 4, all the components in our methods contribute to higher accuracy. Without M1/M2/M3, the network variant suffers notable accuracy drop on most metrics. Moreover, M1 and M3 contribute more significantly than M2. These results clearly demonstrate the effectiveness of each component in our method.

**Table 3:** Top1, Top3, Top5 results achieved by our method with different settings.

Method	CIFAR10			CIFAR100			TinyImageNet		
	Top1	Top3	Top5	Top1	Top3	Top5	Top1	Top3	Top5
M1+M2	83.83%	94.85%	98.22%	48.00%	66.41%	73.27%	43.52%	63.97%	71.79%
M1+M3	92.51%	98.16%	**99.35%**	71.93%	**86.59%**	**90.50%**	73.29%	84.73%	88.41%
M2+M3	79.99%	93.22%	97.41%	64.19%	79.29%	84.63%	72.74%	84.33%	87.30%
Ours (M1+M2+M3)	**92.96%**	**98.32%**	99.33%	**72.17%**	86.55%	90.36%	**73.52%**	**84.95%**	**88.55%**

**Table 4:** AUROC results achieved by our method with different settings.

Method	CIFAR10	CIFAR100	TinyImageNet
M1+M2	97.94%	92.99%	87.03%
M1+M3	99.75%	95.87%	**96.54%**
M2+M3	97.26%	94.70%	96.09%
Ours (M1+M2+M3)	**99.78%**	**96.03%**	96.48%

#### 4.3.2 Effects of different aggregation strategies

We further conduct experiments to study different aggregation strategies. Specifically, we first use predefined weights to aggregate the logits produced by different methods. Then, we study three different weighting strategies, which employ max similarity, inverse entropy, and negative exponential of entropy to fuse logits. As shown in Tables 5 and 6, the method with inverse entropy produces the best performance. Consequently, this strategy is used as the default setting in our experiments.

**Table 5:** Top1, Top3, Top5 accuracy achieved by our method with different weight settings on CIFAR10.

Weighting Method	Top1	Top3	Top5
1:1:1	92.36%	98.22%	99.31%
3:3:4	92.29%	98.21%	99.26%
Max Similarity	92.75%	98.26%	99.34%
Inverse Entropy	**92.96%**	**98.32%**	99.33%
Negative Exponential of Entropy	92.90%	98.31%	**99.36%**

**Table 6:** AUROC results achieved by our method with different weight setting methods on CIFAR10.

Weighting Method	AUROC
1:1:1	99.55%
3:3:4	99.60%
Max Similarity	99.68%
Inverse Entropy	**99.78%**
Negative Exponential of Entropy	99.73%

#### 4.3.3 Quantity and quality of generated reference images

**Fig 3:**
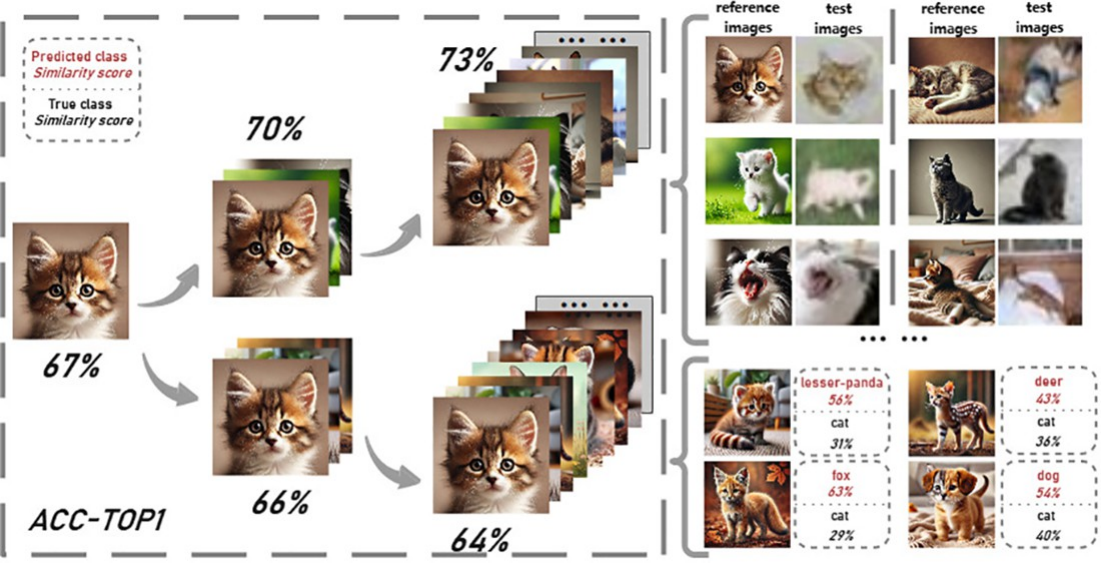
Ablation of the reference images.

Intuitively, the quantity and quality of generated reference images are critical to the final performance. Consequently, we conducted experiments on generating reference images. To increase the diversity of generated images, the object with various realistic poses were generated to depict activities like cats eating, sleeping, and standing. As illustrated in Fig 3, the accuracy (ACC-TOP1) progressively increased from 67% to 73% with more images being generated. This demonstrates large quantity is beneficial to the final accuracy. In addition, we constructed a network variant with only one reference image per category and compared its accuracy with our baseline model with multiple reference images. As shown in Table 7, the model with multiple reference images significantly outperforms the one with a single reference image. This is because additional reference images provide more comprehensive information to describe the properties of each category, ultimately leading to higher accuracy.

**Table 7:** Comparison of Top1 and AUROC results when using one and multiple reference images. The left side of each ‘-’ represents the Top1 result, and the right side of each ‘-’ represents the AUROC result.

Number of Reference Images	CIFAR10	CIFAR100	TinyImageNet
One	88.71%-99.05%	67.78%-96.025%	67.77%-96.00%
Multiple	**92.96%-99.78%**	**72.17%-96.026%**	**73.52%-96.48%**

**Fig 4:**
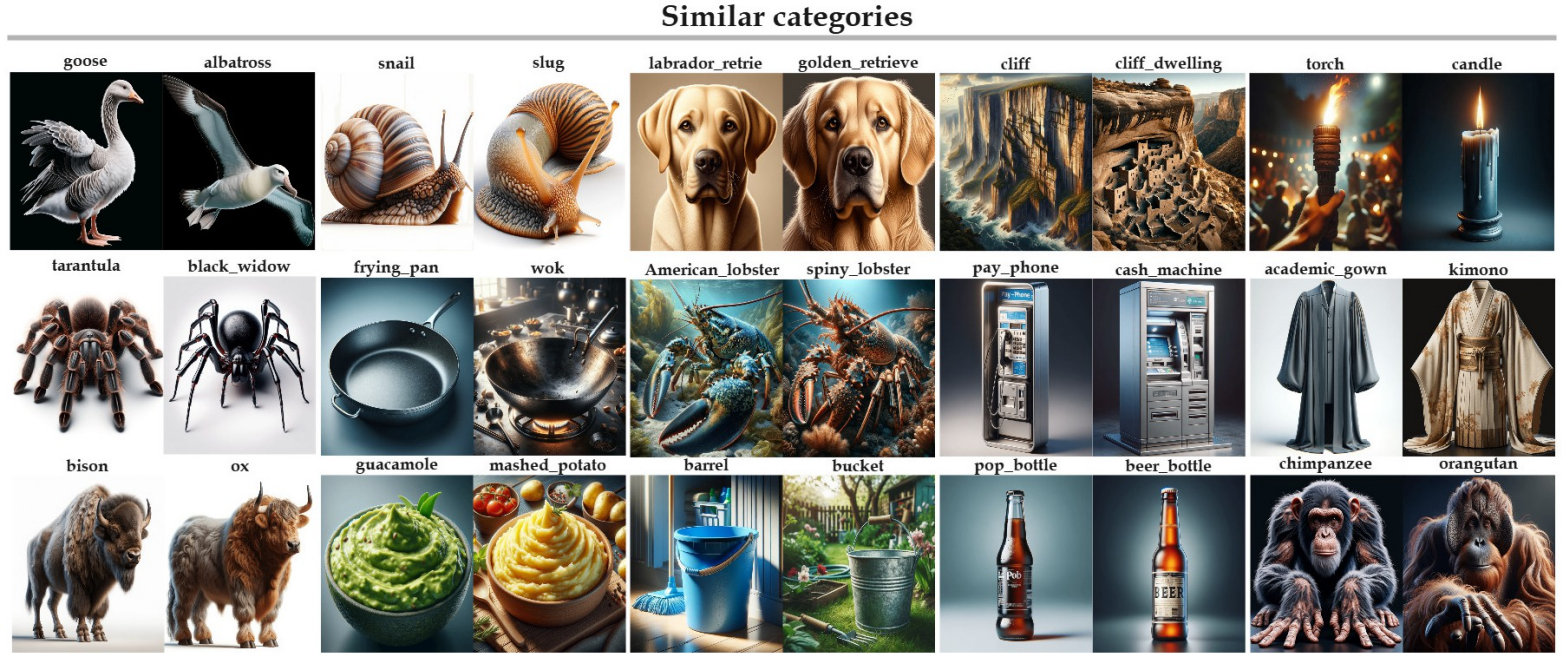
Visualization of synthesized reference images.

On the other hand, as shown in Fig 3, images of low quality may cause confusions between the true category, cat, and other categories like lesser panda, deer, fox, and dog. As a result, ACC-TOP1 is decreased to 64%. This demonstrates that the quality of generated images is also crucial. It can be observed from Fig 4 that the generated hard samples facilitate the model to better distinguish similar categories, thereby achieving higher accuracy.

#### 4.3.4 Visualization results

We first visualize the synthetic images in Fig 4. It can be observed that our method is able to synthesize images for similar categories with similar appearance, which facilitate our method to produce higher accuracy.

**Fig 5:**
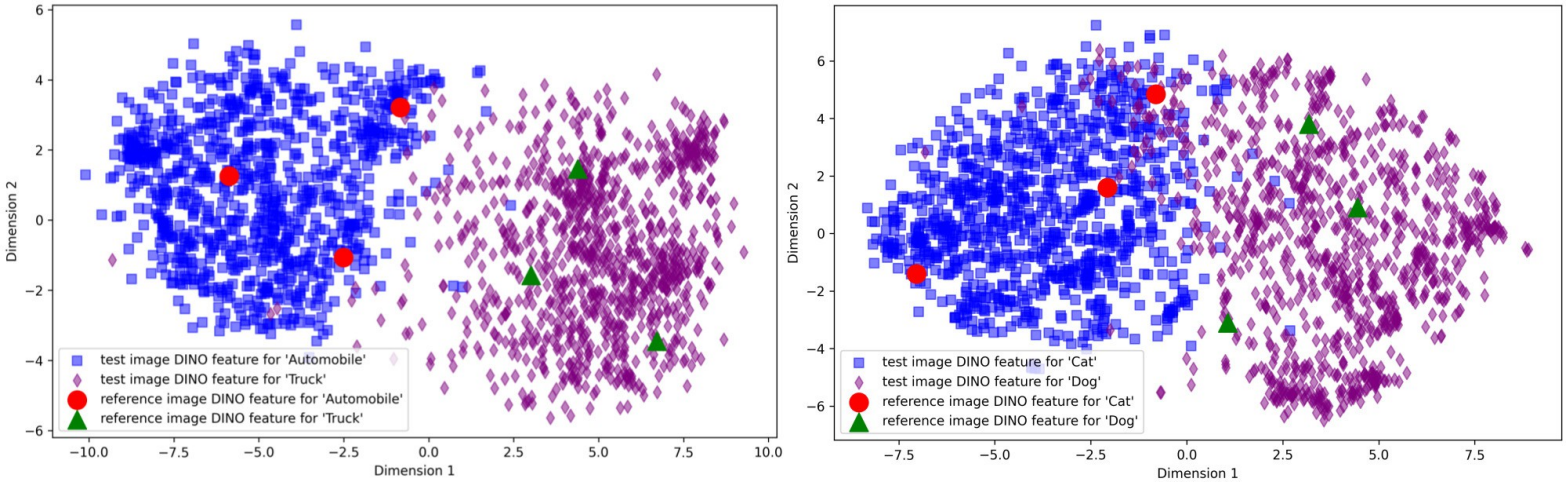
Visualization of the representations for test images and reference images using t-SNE.

We then conduct a detailed analysis for the features of two similar groups of categories [cats, dogs] and [cars, trucks] within the CIFAR-10 dataset. Concretely, for each category, we visualize the features of all test images and the corresponding synthetic images. We employ the t-SNE technique to visualize the representations in Fig 5. As shown in Fig 5, we observe that feature points of synthetic reference images are located at the boundaries between categories, which facilitate the model to distinguish similar categories more clearly.

#### 4.3.5 Analysis of computational complexity and scalability

As shown in Table 8, we present a comprehensive breakdown of the inference time for each stage of our method. Notably, the runtime for steps (1) and (2) is excluded since these steps are conducted using webpage APIs. It is clear that the inference time for steps (3) to (7) is relatively small. For instance, image preprocessing takes approximately 2.1 × 10^−4^ seconds, while feature extraction and classification require 1.3 × 10^−2^ seconds and 2.6 × 10^−2^ seconds, respectively. Overall, processing the entire CIFAR-10 dataset requires approximately 232 seconds, which shows the efficiency of our approach. For larger datasets such as CIFAR-100 and TinyImageNet, the total processing times are approximately 430 seconds and 1970 seconds, respectively, remaining within a reasonable computational range.

**Table 8:** Timeline for zero-shot image classification task on CIFAR-10 dataset.

Step	Description	Time (seconds)
(1)	GPT: generate unknown labels based on known labels	/
(2)	GPT: combine similar categories and feature analysis	/
(3)	DALL-E: generate reference images	15 – 60
(4)	Test-images preprocessing	2.1 × 10^−4^
(5)	CLIP and DINO: Feature extraction	1.3 × 10^−2^
(6)	Classification	2.6 × 10^−2^
(7)	Post-processing	1.2 × 10^−4^

## 5 Conclusion

In this paper, we introduce a framework for zero-shot classification by exploiting knowledge from multiple foundation models. We first employ ChatGPT and DALL-E to synthesize reference images of unseen categories. Then, text-image alignment and image-image alignment are conducted to produce the logits of the test image. Finally, the resultant logits are aggregated to generate the overall prediction. Extensive experimental results on CIFAR-10, CIFAR-100, and TinyImageNet validate the effectiveness of our approach, showcasing its exceptional performance in both open and closed set classification tasks.

Despite promising performance, our method still has some limitations. Specifically, the process of generating synthetic images is relatively computationally complex, especially when dealing with large-scale datasets or real-time applications, which could lead to significant computational overhead. In the future, we will focus on improving the computational efficiency of the image generation process to better fit real-world applications.
